# Neonatal Oral Administration of Chrysin Prevents Long-Term Development of Non-Alcoholic Fatty Liver Disease in a Sexually Dimorphic Manner in Fructose Nurtured Sprague Dawley Rats

**DOI:** 10.3390/life12060790

**Published:** 2022-05-26

**Authors:** Austin A. Ajah, Busisani W. Lembede, Pilani Nkomozepi, Kennedy H. Erlwanger, Trevor T. Nyakudya

**Affiliations:** 1 School of Physiology, Faculty of Health Sciences, University of the Witwatersrand, 7 York Road, Parktown, Johannesburg 2193, South Africa; jlembede@gmail.com (B.W.L.); kennedy.erlwanger@wits.ac.za (K.H.E.); 2 Department of Physiology, Faculty of Basic Medical Sciences, College of Health Sciences, University of Port Harcourt, P.M.B. 5323, Choba, Port Harcourt 500102, Nigeria; 3 Department of Human Anatomy and Physiology, Faculty of Health Sciences, University of Johannesburg, Corner Beit and Siemert Street, Doornfontein, Johannesburg 2094, South Africa; pilanin@uj.ac.za (P.N.); trevor.nyakudya@up.ac.za (T.T.N.); 4 Department of Physiology, School of Medicine, Faculty of Health Sciences, University of Pretoria, Private Bag X323, Gezina, Pretoria 0031, South Africa

**Keywords:** fructose, NAFLD, hepatic lipid, chrysin, neonate

## Abstract

High-fructose diets are linked with the development of non-alcoholic fatty liver disease (NAFLD), the management of which is a burden to society. Interventions with phytochemicals in the early postnatal period may prevent fructose-induced NAFLD later in adulthood. We investigated the protective potential of chrysin against fructose-induced NAFLD. Four-day-old male and female suckling Sprague Dawley rats (N = 112) were randomly grouped and orally gavaged daily with distilled water (negative Control-Cn + W), chrysin(Chr-100 mg/kg), fructose-solution (Fr-20% *w*/*v*), and Chr + Fr between postnatal day (PND) 4 and 21 and then weaned onto normal rat chow and plain drinking water to PND 55. From PND 56 to 130, half of the rats continued on plain water, and the rest had Fr as drinking fluid. Terminally, the liver tissue was collected, and the lipid content was determined and histologically assessed for NAFLD. Dietary Fr induced an increased hepatic lipid content (*p* = 0.0001 vs. Cn + W) both sexes, and it was only attenuated by neonatal Chr in female rats (*p* < 0.05). Histologically, there was increased microvesicular steatosis (*p* = 0.0001 vs. Cn + W) in both sexes, and it was prevented by neonatal Chr (*p* > 0.05). Fr caused macrovesicular steatosis (*p* = 0.01 vs. Cn + W) in females only, and chrysin did not prevent it (*p* > 0.05). Fr induced hepatocellular hypertrophy, and inflammation was observed in females only (*p* = 0.01 vs. Cn + W), and this was prevented by Chr (*p* > 0.05). The collagen area fraction was increased by Fr (*p* = 0.02 (males) and *p* = 0.04 (females) vs. Cn + W, respectively; however, chrysin did not prevent this (*p* > 0.05). Neonatal chrysin prevented some of the deleterious effects of the high-fructose diet on the liver, suggesting that chrysin should be further explored as a strategic prophylactic neonatal intervention against high-fructose-diet-induced NAFLD.

## 1. Introduction

Non-alcoholic fatty liver disease is the excessive accumulation of lipids in the liver without excessive alcohol intake or other factors that could result in liver damage, such as chemicals or infection by hepatitis C virus [1]. Non-alcoholic fatty liver disease is reversible with timely diagnosis and treatment [2]. However, it can progress from simple steatosis to non-alcoholic steatohepatitis (NASH), fibrosis, and possibly hepatocellular carcinoma [3] if untreated. 

The global incidence and prevalence of NAFLD has been estimated to be 25% [4], with the highest prevalence rate in Europe, America, and Asia, at about 24–30% [2], while, in Africa, it is approximately 14% [3]. Management of NAFLD places a heavy burden on healthcare facilities globally [5]. Several causative factors have been linked to the pathogenesis of NAFLD.

Poor lifestyle choices (energy-rich dietary intake and physical inactivity); and genetic, epigenetic, and environmental factors, especially during the early life of an organism, were identified as major factors in the development of NAFLD [6]. Excessive fructose intake, particularly, has been reported to cause an increase in the incidence and prevalence of obesity and NAFLD [7]. Fructose is highly lipogenic as a result of its metabolism not being regulated in the liver by escaping the rate-limiting process of glycolysis [8] (Taskinen et al., 2019). Hence, its consumption increases the lipid content of the liver, and this can manifest as NAFLD [9]. Previously, it has been established in our lab that 20% fructose can induce NAFLD in neonatal rats [10]; hence, this necessitated the choice.

Previously the two-hit hypothesis was highly favored in the explanation of NAFLD presentation, progression, and severity [11]: that exposure (hit) to triggering factors could occur at different times in life, resulting in disease. In this study, the first hit refers to the administration of high-fructose diets to the rats either during the neonatal (early hit) or adult (late hit) phase, while the second hit refers to additional fructose intake during the adult stage. Thus, a double hit refers to the administration of fructose during both the neonatal and adult phases. In the case of the two-hit hypothesis for NAFLD, the first hit triggers a reversible excessive accumulation of the lipid droplets in the liver [12]. The second hit then brings about the severity of this condition, translating to liver inflammation and necrosis due to steatosis [12]. 

However, recent studies have shown that NAFLD can occur without progressive hits [13]. An alteration in gut microbiome (dysbiosis) has also been attributed to the pathophysiology of NAFLD [14]. 

Lifestyle changes such as increasing physical exercise, dietary modifications, and weight loss are effective in the management and treatment of NAFLD [4]. However, there is no specific approved pharmacologic intervention for the management of NAFLD, as various treatment options deal with individual components of NAFLD [15,16]. Hence, an alternative and complementary approach, such as the use of medicinal plants and their constituent phytochemicals, must be considered in the management and treatment of NAFLD. Chrysin has been reported to possess anti-inflammatory, anti-diabetic, and anti-obesogenic properties [17,18]. However, most of the beneficial effects of chrysin were reported in adult animal models, and there is scarce information on neonatal animal models. The aforementioned beneficial biological properties of chrysin make it a potential bioactive flavonoid candidate that can be orally administered during suckling to potentially program for protection against the subsequent development of diet-induced NAFLD later on in adulthood [19]. Rats are altricial species that depend solely on the dams for milk as a source of nourishment during the neonatal periods of life [20]. The suckling period of rats is equivalent to the last trimester of gestational development in humans [21,22]. The neonatal period is characterized by rapid development and differentiation of various organs and physiological systems [20]. Thus, dietary interventions during the neonatal period can impact on the development and function of organs and physiological systems through epigenetic changes [20]. This can translate into increased susceptibility or protection against the development of NAFLD in adulthood [20]. Ideally, the prevention of disease is better than the cure. Hence, this study sought to interrogate the potential protective effect of orally administered chrysin in the neonatal period against early or late ‘single-hit’ and ‘double-hit’ high-fructose-diet-induced non-alcoholic fatty liver disease in adult male and female Sprague Dawley rats.

## 2. Materials and Methods

### 2.1. Study Settings 

This study was carried out at the Wits Research Animal Facility (WRAF) of University of the Witwatersrand, Johannesburg, South Africa. 

### 2.2. Ethical Clearance

Ethical clearance for the study was granted by the University of the Witwatersrand’s Animal Research Ethics Committee (ethical clearance number: 2019/07/042B).

### 2.3. Animals and Housing

One hundred and twelve (112) 4-day-old suckling male and female Sprague Dawley rat pups from 13 nulliparous female dams were used for this study. Dams with litters between 8 and 12 pups were used. During the pre-weaning period, from postnatal day (PND) 4 to 21, the rat litters were housed with their respective dams in the WRAF (University of the Witwatersrand). After weaning, from PND 21 onward, the rats were housed individually and allowed ad libitum access to feed and drinking fluid. The room temperature was maintained at a range of 24 ± 2 °C, and a 12 h dark-and-light cycle (7:00 h to 19:00 h) was adhered to throughout the experimental treatments.

### 2.4. Experimental Design

**Figure 1:**
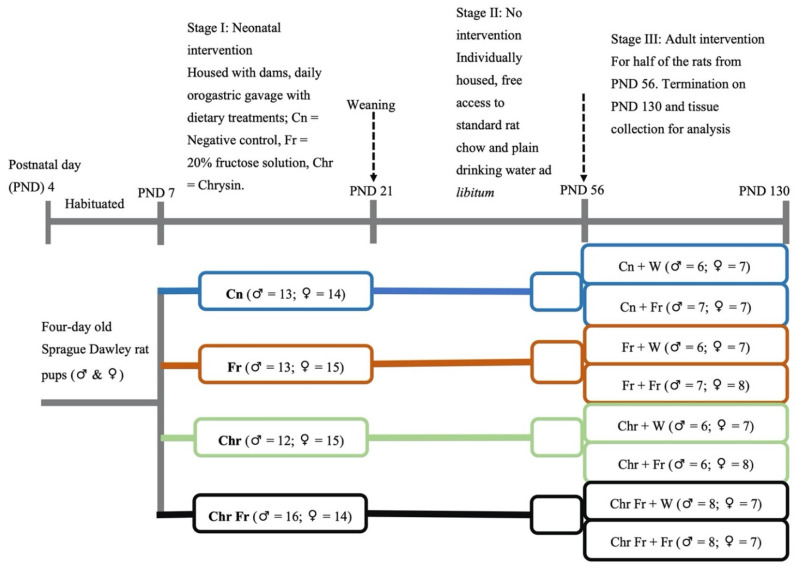
Schematic diagram of the experimental design and rat allocations. Cn = negative control; Fr = fructose; Chr = chrysin; Chr Fr = chrysin + fructose; ♂ = male rats; ♀ = female rats.

This interventional study was divided into three experimental stages: neonatal, no intervention (transition into the adult stage), and adult intervention stages. A summary of the group allocations and timelines is shown in Figure 1. The first experimental stage, the ‘neonatal intervention’ stage, was from PND 4 to 21 (16 days), during which some the neonatal rats received the first dietary insult with a high-fructose diet. This stage of intervention is termed as the early hit. The second stage was the ‘no intervention’ stage and commenced from PND 22 to 56 (5 weeks). The third stage was the ‘adult intervention’ stage, and it commenced from PND 56 continuing to 130 (11 weeks), during which half of the adult rats received a high-fructose diet, while the other half did not. Therefore, some rats were fed a high-fructose diet in both the neonatal and adult phases (double hit), while those that were administered with a dietary insult either in the neonatal or the adult phase are described as receiving an early or late single hit, respectively.

On postnatal day (PND) 4, the pups and dams were obtained and acclimatized to handling and weighing up to PND 6, before the commencement of experimental treatments. The rat pups were randomly allocated to four treatment groups on PND 7. Pups were randomly allocated to the groups in a split-litter fashion and differentiated by marking on their tails, using non-toxic permanent ink. The rat pups within each litter were orally gavaged once daily in the morning (08:00–09:00) via orogastric tube mounted on a 1 mL syringe for 14 consecutive days from PND 7–20, with the following treatments: 

**Group I**—10 mL/kg of 0.5% DMSO (Cn) vehicle (negative) control; **Group II**—10 mL/kg of 20% *w*/*v* fructose solution (Fr) first (early) hit group dissolved in 0.5% DMSO; **Group III**—10 mL/kg of 100 mg/kg chrysin (Chr) dissolved in 0.5% DMSO chrysin treated group; and **Group IV**—10 mL/kg of Fr + Chr in 0.5% DMSO first (early) hit chrysin treatment group.

The dose of chrysin that was used in the current study is similar to that which has previously been used in a different study [23] Satyanarayana et al. (2015)

The second stage was the ‘no intervention’ stage. On PND 21, the pups were weaned onto standard rat chow and allowed to transition into the adult phase in a one-rat-to-a-single-cage fashion. Rats were allowed access to food and water ad libitum, as they were left untreated from PND 22 to 55.

The third stage was the ‘adult intervention’ stage. On PND 56, half of the rats initially on intervention in stage one were allocated to a high-fructose diet (20% fructose solution), and the rest remained on plain drinking water until PND 130 viz:

**Group I**—Cn + W [(plain drinking water (negative)] control; **Group II**—Cn + Fr (Neonatal control with late single hit high-fructose diet in adulthood); **Group III**—Fr + W [(early (neonatal) single hit of high-fructose diet with plain drinking water in adulthood)]; **Group IV**—Fr + Fr [(double (neonatal and adulthood) hit of high-fructose diet)]; **Group V**—Chr + W [(neonatal chrysin with plain drinking water (negative) control]; **Group VI**—Chr + Fr [(late (neonatal) single hit of high-fructose diet with neonatal chrysin treatment group)]; **Group VII**—Chr Fr + W [(early (neonatal) single hit of high-fructose diet in combination with neonatal chrysin treatment group)]; and **Group VIII**—Chr Fr + Fr (double hit of high-fructose diet in combination with neonatal chrysin treatment group).

### 2.5. Measurement of Health Outcomes Associated with NAFLD

#### 2.5.1. Body Mass 

In stage I, pups were weighed daily with an electronic scale (Snowrex Electronic Scale, Clover Scales, Johannesburg, South Africa) to determine growth performance and to maintain a constant daily dosage for the treatment regimen with respect to body mass during the 15 days’ treatment period. In stages II and III, the rats were weighed twice a week in order to monitor their growth and general health status.

#### 2.5.2. Terminal Procedures

On postnatal day 130, following an overnight fast, the rats’ terminal body masses were measured, and the rats were then euthanized by intraperitoneal injection of 150 mg/kg sodium pentobarbital (Eutha-naze^®^, Bayer, Johannesburg, South Africa). 

The liver of each rat was carefully dissected out and weighed on an electronic scale (Presica 310 M, Presica Instruments, Dietikon, Switzerland). The hepatosomatic index was computed by dividing the mass of each liver by the respective terminal body mass of each rat and expressed as a percentage (%).

Calculation of the hepatosomatic index (%):

$$\mathrm{Hepatosomatic}\;\mathrm{index}\;\left(\%\right)=\frac{\mathrm{Mass}\;\mathrm{of}\;\mathrm{the}\;\mathrm{liver}\;\left(g\right)}{\mathrm{Terminal}\;\mathrm{body}\;\mathrm{mass}\;\left(g\right)}\;\;\times \;100$$

A sample from the right lobe of each liver was preserved in 10% phosphate buffered formalin solution (Merck, Johannesburg, South Africa) for histological analysis. The rest of the liver was stored in sealed Ziploc plastic bags at −20 °C for total lipid content determination.

#### 2.5.3. Sample Processing for Histology

Preserved samples of liver tissues were processed for histological analysis, using an automated tissue processor (Microm STP 120 Thermo Scientific, Waltham, MA, USA), and embedded in paraffin wax. The liver samples were then sectioned at 5 µm, using a microtome (Leica instruments GmbH, (Pty) Ltd., Wetzlar, Germany), and then mounted on glass slides which were stained automatically with hematoxylin and eosin (H&E) or Mason’s trichrome (MT) in a Gemini AS slide stainer coupled to a Clearvue coverslipper (Thermo Scientific, Massachusetts, USA). The liver H&E-stained slides were used to ascertain hepatocellular changes, while the MT stain was used to assess collagen deposition as a marker of fibrosis.

The H&E- and MT-stained liver slides were viewed under a light microscope (Olympus XC 10 HD, Glasgow, UK) mounted on an Olympus BH2-RFCA camera and (Carl Zeiss Microscopy GmbH, Göttingen, Germany), respectively, to evaluate the histological changes in the liver.

#### 2.5.4. Liver Histology and Scoring for Non-Alcoholic Fatty Liver Disease 

The progression and severity of fatty liver disease was assessed by scoring the H&E-stained liver sections, using semi-quantitative non-alcoholic fatty liver disease scoring criteria for rodents [24]. Macrosteatosis, microsteatosis, and hepatocellular hypertrophy were graded with scores 0, 1, 2, and 3, representing <5%, 5–33%, 33–66%, and >66% of liver mass affected, respectively. In addition, Lobular inflammation was scored 0, 1, 2, and 3, representing <0.5, 0.5–1, 1–2, and >2 foci in the liver section, respectively. Scoring was performed blindly by a histologist (PN) in order to avoid bias.

The MT-stained liver sections were quantitatively assessed for collagen deposition at ×40 magnification, as described by Ibrahim et al. (2019) [25], using ImageJ (ImageJ, version 1.51, Bethesda, ML, USA). The area (*A*) and area fraction (*A_fraction_*) of each liver section (40×) occupied by connective tissue were measured by using the point-counting method and computed by using Equations (1) and (2):

(1) A=ap×Σp

(2) $$A_{fraction}=A÷0.149\;{\mathrm{mm}}^{2}\times 100$$

where *ap* is the area per point (0.002 mm^2^), and *∑p* is the sum of the points falling on the connective tissue within a camera field (0.149 mm^2^) of each liver section. A total of 20 camera fields were used for each section.

#### 2.5.5. Total Liver Lipid Content Determination 

The total liver lipid content was determined by the Soxhlet ether-solvent method of lipid extraction, as described by AOAC (2005; method number 920.39) [26]. Briefly, stored frozen liver samples were placed into a benchtop freeze dryer (VirTis benchtop freeze dryer, SP Industries Inc., New York, NY, USA) and lyophilized for 24 h and then finely ground. Thereafter, 0.5 g of each sample was placed on fat-free cotton wool in a cellulose thimble and transferred into a Soxhlet extraction chamber. For each sample, an empty distillation (round bottomed) flask was weighed, its mass was recorded as (M_1_), and 200 mL of petroleum ether was dispensed into it. The distillation flask and its content were then placed onto the heating pad of the mantle, and the thermostat was set at the boiling point of petroleum ether (50 ± 10 °C). The extraction was performed for two hours. The collection flask was transferred to a water bath set at (50 ± 10 °C) and connected to a rotary evaporator attached to a vacuum pump for the removal of the petroleum ether, leaving the oil in the flask. When the flask cooled to room temperature it was re-weighed, and the second mass (M_2_) was recorded. The percentage of fat in the dried liver sample was then calculated by using the following formulas:

Calculation of the extracted fat (%):

$$\%\;\mathrm{fat}\;=\frac{\mathrm{Weight}\;\mathrm{of}\;\mathrm{flask}\;\mathrm{with}\;\mathrm{oil}\;\left(M2\right)-\mathrm{Weight}\;\mathrm{of}\;\mathrm{empty}\;\mathrm{flask}\;\left(M1\right)}{\mathrm{Weight}\;\mathrm{of}\;\mathrm{liver}\;\mathrm{sample}\;\left(0.5\;g\right)}\;\;\times \;100$$

## 3. Statistical Analysis

Data were analyzed using GraphPad Prism 8 software (Graph-pad Software Inc., San Diego, CA, USA). Parametric data were expressed as mean ± SD and non-parametric data [non-alcoholic fatty liver disease activity score (NAS)] as median and interquartile range (min, max) and frequency. Multiple-group parametric data were analyzed by one-way analysis of variance (ANOVA), followed by a Tukey’s post hoc test for multiple group comparisons. The Kruskal–Wallis test (non-parametric one-way ANOVA was used to analyse multiple-group NAS data followed by a multiple-comparisons Dunns post hoc test to compare medians. Sex variations were analyzed using two-way ANOVA. Data was considered statistically significant when *p* ≤ 0.05.

## 4. Results

### 4.1. Hepatosomatic Index (Relative %BM) and Percentage (%) Lipid Content of Liver of Male and Female Rats

- Hepatosomatic index of male rats

**Figure 2:**
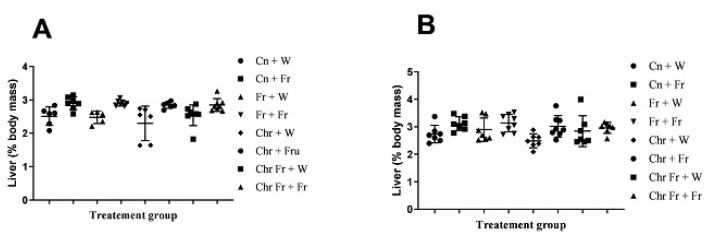

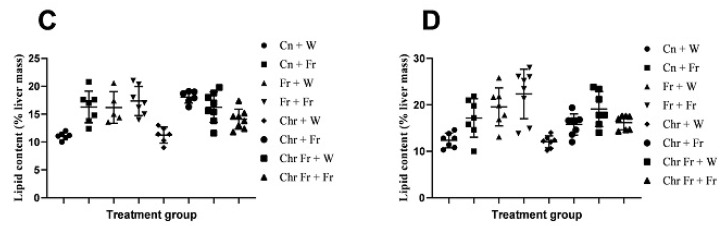
Effect of neonatal oral administration of chrysin on the hepatosomatic indices and percentage (%) lipid content of the liver of male (**A**,**C**) and female (**B**,**D**) rats fed high-fructose diets. Cn + W = administered water during neonatal phase and water during adult phase (M = 6, F = 7); Cn + Fr = administered water during neonatal phase and fructose during adult phase (M = 7, F = 7); Fr + W = administered fructose during neonatal phase and water during adult phase (M = 5, F = 7); Fr + Fr = administered fructose during neonatal phase and fructose during adult phase (M = 7, F = 8); Chr + W = administered chrysin during neonatal phase and water during adult phase (M = 6, F = 7); Chr + Fr = administered chrysin during neonatal phase and fructose during adult phase (M = 6, F = 8); Chr Fr + W = administered water and chrysin during neonatal phase and water during adult phase (M = 8, F = 7); Chr Fr + Fr = administered chrysin and fructose during neonatal phase and fructose during adult phase (M = 8, F = 7); M = males; F = females.

Male rats which were administered with a double-hit high-fructose diet neonatally and in adulthood (Fr + Fr, *p* = 0.0030), late single-hit high-fructose diet (Cn + Fr, *p* = 0.0033), neonatal chrysin administration and a late single-hit high-fructose diet intake in adulthood (Chr + Fr, *p* = 0.02), and neonatal chrysin administration and double-hit high-fructose diet intake in adulthood (Chr Fr + Fr, *p* = 0.0073) resulted in a significant higher relative masses of the liver when compared to neonatal chrysin administration (Chr + W), respectively, in male rats (Figure 2A). In summary, the high-fructose diet (both single-hit and double-hit) caused an increase in relative masses of the liver of male rats, and the neonatal administration of chrysin did not prevent the increase.

b. Hepatosomatic index of female rats

Female rats administered chrysin neonatally without fructose (Chr + Fr) had significantly lower hepatosomatic indices compared to those which had the double-hit high-fructose diet neonatally and in adulthood (Fr + Fr) and late single-hit high-fructose diet (Cn + Fr) (*p* = 0.04 and *p* = 0.02, respectively), as seen in Figure 2B. However, the relative masses of the liver were similar (*p* > 0.05) for female rats in the rest of the treatment groups (Figure 2B). In summary, the high-fructose diet (both single-hit and double-hit) caused an increase in relative masses of the liver of female rats, and the neonatal administration of chrysin did not prevent the increase.

Overall, both male and female rats had similar relative masses of the liver thus (main sex effects (*p* = 0.4554)). However, there were significant treatment (*p* = 0.0004) and interaction (*p* = 0.0002) effects.

c. Hepatic lipid content in male rats

The male rats that were administered a double-hit (Fr + Fr) high-fructose diet had a significantly increased percentage of hepatic lipid content compared to the negative control and rats administered chrysin neonatally (*p* = 0.0001 vs. Cn + W; *p* = 0.0002 vs. Chr + W), as shown in Figure 2C. Administration of high-fructose diets as a late single hit (D + Fr) also caused a significant increase (*p* = 0.002 vs. Cn + W; *p* = 0.003 vs. Chr + W) in the percentage of hepatic lipid content of male rats (Figure 2C). Similarly, the early single-hit (Fr + W) high-fructose diet caused an increase (*p* = 0.007 vs. Cn + W; *p* = 0.01 vs. Chr + W) in the percentage of hepatic lipid content of male rats (Figure 2C). Furthermore, a double-hit high-fructose diet in combination with neonatal chrysin (Chr Fr + Fr) significantly increased (*p* = 0.0001 vs. Cn + W) the percentage of hepatic lipid content of male rats (Figure 2C). Moreover, an early single-hit high-fructose diet in combination with neonatal chrysin (Chr Fr + W) significantly increased (*p* = 0.002 vs. Cn + W; 0.002 vs. Chr + W) the percentage of hepatic lipid content of male rats (Figure 2C). Finally, a late single-hit high-fructose diet in combination with neonatal chrysin (Chr + Fr) significantly increased (*p* = 0.03 vs. Chr Fr + Fr) the percentage of hepatic lipid content of male rats (Figure 2C). In summary, the high-fructose diet (single-hit and double-hit) caused an increase in the percentage lipid content of the liver of male rats, and neonatal chrysin administration could not prevent this increase.

d. Hepatic lipid content in female rats

There was a significant increase in hepatic lipid content of female rats that received early single-hit high-fructose diet without neonatal chrysin (Fr + W, *p* = 0.005), double-hit high-fructose diet without neonatal chrysin (Fr + Fr, *p* = 0.0001), and early single-hit high-fructose diet with neonatal chrysin (Chr Fr + W, *p* = 0.01) when compared to the negative control (Cn + W; Figure 2D). Finally, the neonatal administration of chrysin (Chr Fr + Fr) attenuated (*p* > 0.05) the increase in percentage of hepatic lipid content induced by the double-hit high-fructose diet (Figure 2D). In summary, the high-fructose diet (both single-hit and double-hit) caused an increase in the percentage of hepatic lipid content of female rats, and the administration of neonatal chrysin attenuated this increase only in the double-hit high-fructose diet. 

Overall, male rats had a significantly lower hepatic lipid content than female rats (main sex effects (*p* < 0.0021), treatment (*p* = 0.0001). However, there was no significant interaction between both sexes (*p* = 0.0654).

### 4.2. Liver Histopathology

- Microvesicular steatosis in male rats

**Figure 3:**
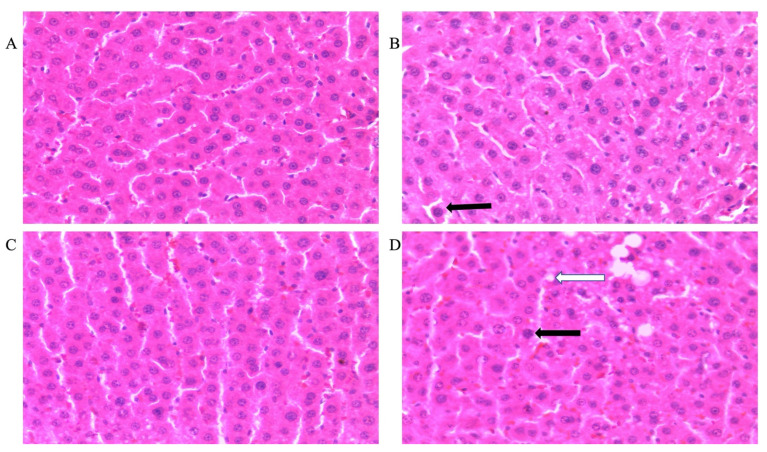

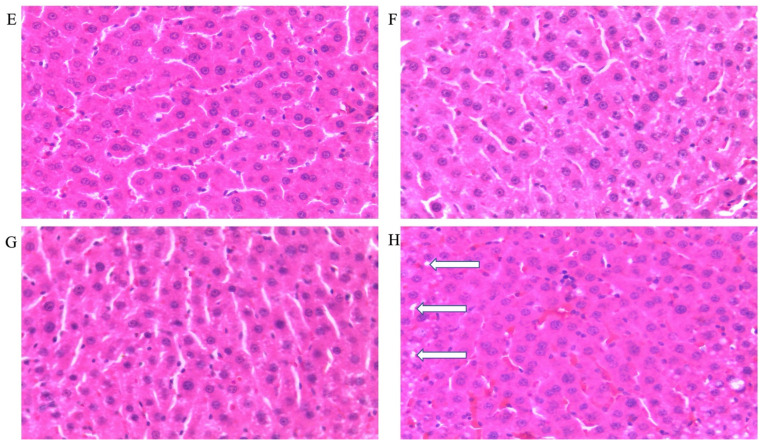
Photomicrographs showing histopathological features after hematoxylin and eosin staining of liver cross-sections from a representative male rat of each experimental group (**A** = Cn + W; **B** = Cn + Fr; **C** = Fr + W; **D** = Fr + Fr; **E** = Chr + W; **F** = Chr + Fr; **G** = Chr Fr + W; **H** = Chr Fr + Fr). Solid black arrows indicate aggregate of inflammatory cells. The white arrows indicate microvesicular steatosis. Magnification = 40×.

Figure 3 shows representative photomicrographs of the liver histology from each of the experimental groups (H&E stain) of male rats. There was a notable fatty infiltration in the sections represented in Figure 3D,H.

**Figure 4:**
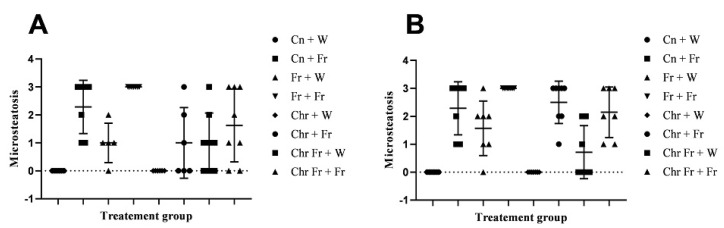

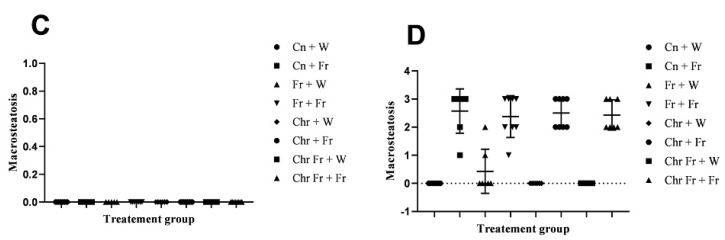
Effect of neonatal oral administration of chrysin on microsteatosis and macrosteatosis in the liver of male (**A**,**C**) and female (**B**,**D**) rats fed a high-fructose diet.

Double-hit (Fr + Fr) high-fructose diets significantly increased (*p* = 0.0007 vs. Cn + W; *p* = 0.0007 vs. Chr + W) microvesicular steatosis in the liver of male rats (Figure 3D,H and Figure 4A). Similarly, late single-hit (Cn + Fr) high-fructose diets significantly increased (*p* = 0.02 vs. Cn + W; *p* = 0.02 vs. Chr + W) microvesicular steatosis in the liver of male rats (Figure 3D,H and Figure 4A). The neonatal administration of chrysin (Chr + FR and Chr Fr + Fr) prevented (*p* > 0.05) the increase in microvesicular steatosis induced by the late single-hit and double-hit high-fructose diet (Cn + Fr and Fr + Fr), respectively (Figure 4A).

b. Microvesicular steatosis in female rats

**Figure 5:**
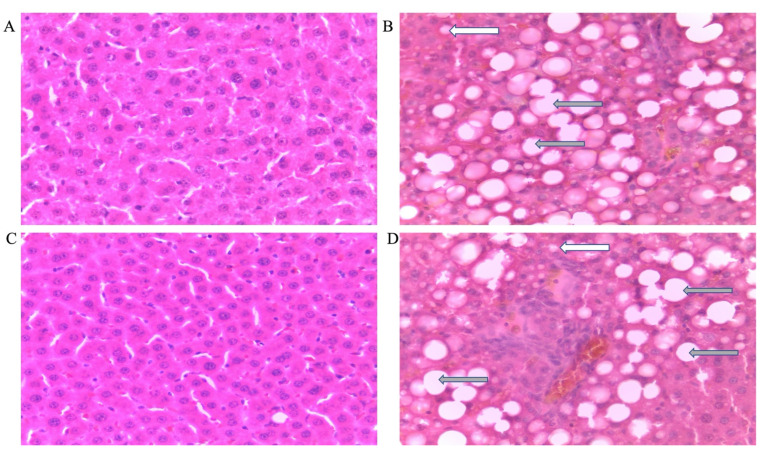

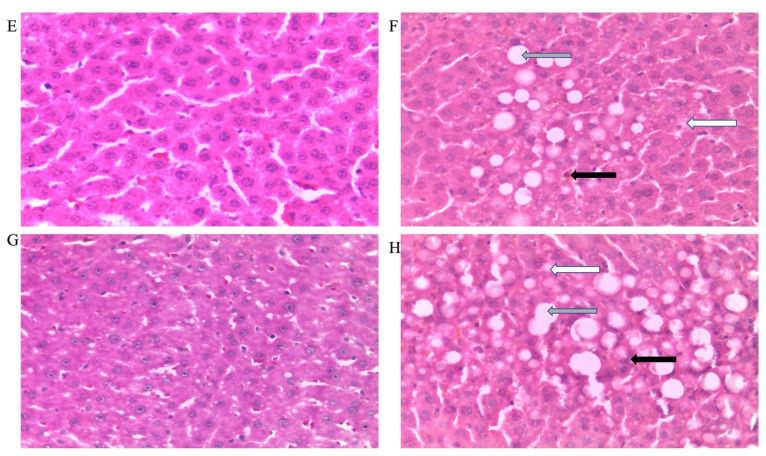
Photomicrographs showing histopathological features after hematoxylin and eosin staining of liver cross-sections from a representative female rat of each experimental group (**A** = Cn + W; **B** = Cn + Fr; **C** = Fr + W; **D** = Fr + Fr; **E** = Chr + W; **F** = Chr + Fr; **G** = Chr Fr + W; **H** = Chr Fr + Fr). Solid black arrows indicate aggregate of inflammatory cells. The white arrows indicate microvesicular steatosis. Gray arrows indicate macrovesicular steatosis. Magnification = 40×.

Figure 5 shows representative photomicrographs of female rats’ liver histology from each experimental group (H&E stain). As shown in Figure 5B,D,F,H, there was a significant fatty infiltration (macro-vesicular steatosis) in the sections of the fructose-only treated group that was generally absent in the other treatment groups. The double-hit (Fr + Fr) high-fructose diets significantly increased (*p* = 0.0001 vs. Cn + W; *p* = 0.0001 vs. Chr + W; *p* = 0.01 vs. Chr Fr + W) microvesicular steatosis in the liver of female rats across the various groups (Figure 4B and Figure 5). Similarly, late single-hit (Cn + Fr) high-fructose diets significantly increased (*p* = 0.02 vs. Cn + W; *p* = 0.02 vs. Chr + W) microvesicular steatosis in the liver of female rats across the various groups (Figure 4B and Figure 5). The neonatal administration of chrysin (Chr Fr + Fr) prevented (*p* > 0.05) the increase in microvesicular steatosis induced by the double-hit high-fructose diet (Fr + Fr; Figure 4B).

Overall, there was no significant difference in the microvesicular steatosis in the liver of male and female rats: main sex effects (*p* = 0.0629) and their interaction (*p* = 0.1066). However, there was a significant treatment effect (*p* < 0.0001).

c. Macrovesicular steatosis in male rats

There were no significant differences (*p* > 0.05) in macrovesicular steatosis scores for the livers of male rats across the various groups (Figure 4C).

d. Macrovesicular steatosis in female rats

**Figure 6:**
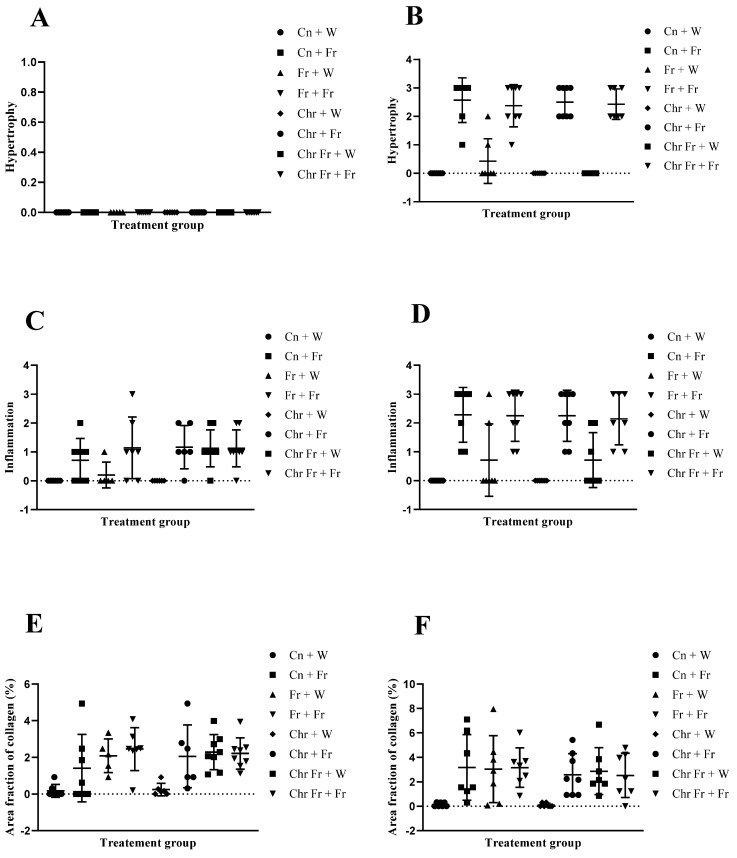
Effect of neonatal oral administration of chrysin on hypertrophy, inflammation, and area fraction of collagen in the liver of male (**A**,**C**,**E**) and female (**B**,**D**,**F**) rats fed a high-fructose diet.

Figure 6 show representative photomicrographs of the liver histology from each of the experimental groups (H&E stain) of female rats. As shown in Figure 5B,D,F,H, there was a significant fatty infiltration (macrovesicular steatosis) in the sections of the fructose–only treated group that was generally absent in the other treatment groups.

The double-hit (Fr + Fr) high-fructose diets significantly increased (*p* = 0.01 vs. Cn + W; *p* = 0.01 vs. Chr + W; *p* = 0.01 vs. Chr Fr + W) macrovesicular steatosis in the liver of female rats across the various groups (Figure 4D and Figure 5). Similarly, late single-hit (Cn + Fr) high-fructose diets significantly increased (*p* = 0.005 vs. Cn + W; *p* = 0.04 vs. Fr + W; *p* = 0.005 vs. Chr + W; *p* = 0.005 vs. Chr Fr + W) macrovesicular steatosis in the liver of female rats across the various groups (Figure 4D and Figure 5). Furthermore, late single-hit high-fructose diets in combination with neonatal chrysin (Chr + Fr) significantly increased (*p* = 0.008 vs. Cn + W; *p* = 0.008 vs. Chr + W; *p* = 0.008 vs. Chr Fr + W) macrovesicular steatosis in the liver of female rats across the various groups (Figure 4D and Figure 5). Finally, double-hit high-fructose diets in combination with neonatal chrysin (Chr Fr + Fr) significantly increased (*p* = 0.02 vs. Cn + W; *p* = 0.03 vs. Chr + W; *p* = 0.02 vs. Chr Fr + W) macrovesicular steatosis in the liver of female rats across the various groups (Figure 4D and Figure 5). In summary, the high-fructose diet (both single-hit and double-hit) caused a significant increase (*p* < 0.0001) in hepatic macrovesicular steatosis, and neonatal chrysin did not prevent the increase.

Overall, male rats had significantly lower macrovesicular steatosis scores than female rats: main sex effects (*p* < 0.0001), treatment (*p* < 0.0001) and their interaction (*p* < 0.0001).

e. Hypertrophy in male rats

There was no significant difference (*p* > 0.05) in hepatocellular hypertrophy scores for the livers of male rats across the various groups (Figure 6A).

f. Hypertrophy in female rats

Administration of double-hit (Fr + Fr) high-fructose diets significantly increased (*p* = 0.01 vs. Cn + W; *p* = 0.01 vs. Chr + W; *p* = 0.01 vs. Chr Fr + W) hypertrophy in the liver of female rats across the various groups (Figure 5 and Figure 6B). Similarly, late single-hit (Cn + Fr) high-fructose diets significantly increased (*p* = 0.005 vs. Cn + W; *p* = 0.04 vs. Fr + W; *p* = 0.005 vs. Chr + W; *p* = 0.005 vs. Chr Fr + W) hypertrophy in the liver of female rats across the various groups (Figure 5 and Figure 6B). Finally, late single-hit high-fructose diets in combination with neonatal chrysin (Chr + Fr) significantly increased (*p* = 0.008 vs. Cn + W; *p* = 0.008 vs. Chr + W; *p* = 0.008 vs. Chr Fr + W) hypertrophy in the liver of female rats across the various groups (Figure 5 and Figure 6B). However, neonatal administration of chrysin (Chr Fr + Fr) prevented (*p* > 0.05) the increase in hypertrophy induced by the double-hit high-fructose diet (Fr + Fr; Figure 6B). In summary, the high-fructose diet (both late single-hit and double-hit) caused a significant increase (*p* < 0.0001) in hepatocellular hypertrophy, and neonatal administration chrysin prevented it.

Overall, male rats had significantly lower hepatocellular hypertrophy scores than female rats: main sex effects (*p* < 0.0001), treatment (*p* < 0.0001), and their interaction (*p* < 0.0001).

g. Inflammation in male rats

There was no significant difference (*p* > 0.05) in inflammation scores for the livers of male rats across the various groups (Figure 3 and Figure 6C).

h. Inflammation in female rats

Administration of double-hit (Fr + Fr) high-fructose diets significantly increased (*p* = 0.01 vs. Cn + W; *p* = 0.01 vs. Chr + W) inflammation in the liver of female rats across the various groups (Figure 5 and Figure 6D). Similarly, late single-hit (Cn + Fr) high-fructose diets significantly increased (*p* = 0.01 vs. Cn + W; *p* = 0.01 vs. Chr + W) inflammation in the liver of female rats across the various groups (Figure 5 and Figure 6D). Finally, late single hit high-fructose diets in combination with neonatal chrysin (Chr + Fr) significantly increased (*p* = 0.01 vs. Cn + W; *p* = 0.01 vs. Chr + W) inflammation in the liver of female rats across the various groups (Figure 5 and Figure 6D). However, neonatal administration of chrysin (Chr Fr + Fr) prevented (*p* > 0.05) the increase in inflammation induced by the double-hit high-fructose diet (Fr + Fr; Figure 6D). In summary, the high-fructose diet (late single-hit and double-hit) caused a significant increase (*p* < 0.0001) in inflammation, of which only the double-hit inflammatory response was prevented by neonatal administration of chrysin.

Overall, male rats had significantly lower hepatocellular inflammation scores than female rats: main sex effects (*p* < 0.0001), treatment (*p* < 0.0001), and their interaction (*p* = 0.0089).

i. Area fraction of collagen in male rats

**Figure 7:**
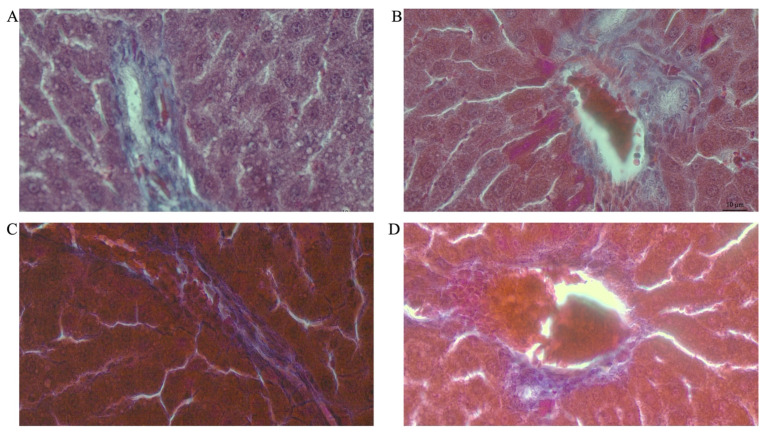

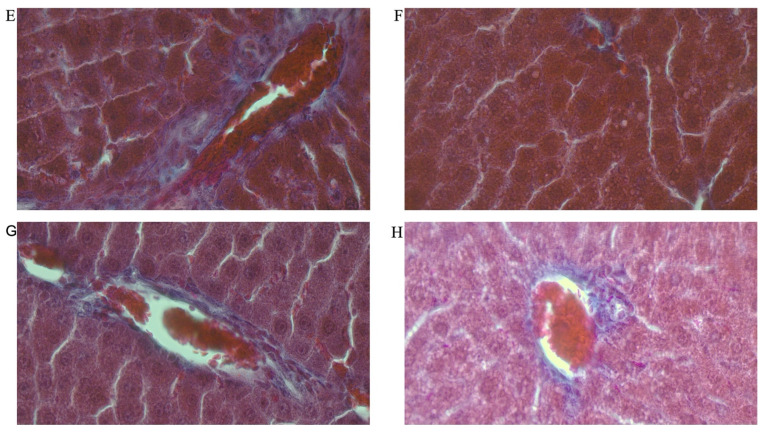
Photomicrographs showing histopathological features after Mason’s trichrome (MT) staining of liver cross-sections from a representative male rat of each experimental group (**A** = Cn + W; **B** = Cn + Fr; **C** = Fr + W; **D** = Fr + Fr; **E** = Chr + W; **F** = Chr + Fr; **G** = Chr Fr + W; **H** = Chr Fr + Fr). Magnification = 40×.

Figure 7A–H shows representative photomicrographs of the liver histology from each of the experimental groups (MT stain) of male rats.

Double-hit (Fr + Fr) high-fructose diets significantly increased (*p* = 0.01 vs. Cn + W; *p* = 0.04 vs. Chr + W) collagen area fraction in the livers of male rats (Figure 6E and Figure 7). Moreover, the double-hit high-fructose diet with neonatal chrysin (Chr Fr + Fr) significantly increased (*p* = 0.03 vs. Cn + W; *p* = 0.04 vs. Chr + W) collagen area fraction in the liver of male rats (Figure 6E and Figure 7). Finally, an early single-hit high-fructose diet and neonatal chrysin (Chr Fr + W) significantly increased (*p* = 0.02 vs. Cn + W; *p* = 0.03 vs. Chr + W) collagen area fraction in the liver of male rats (Figure 6E and Figure 7). In summary, the high-fructose diet (both single-hit and double-hit) caused a significant increase (*p* < 0.001) in collagen area fraction in the liver of male rats, and neonatal chrysin did not prevent the increase.

j. Area fraction of collagen in female rats

**Figure 8:**
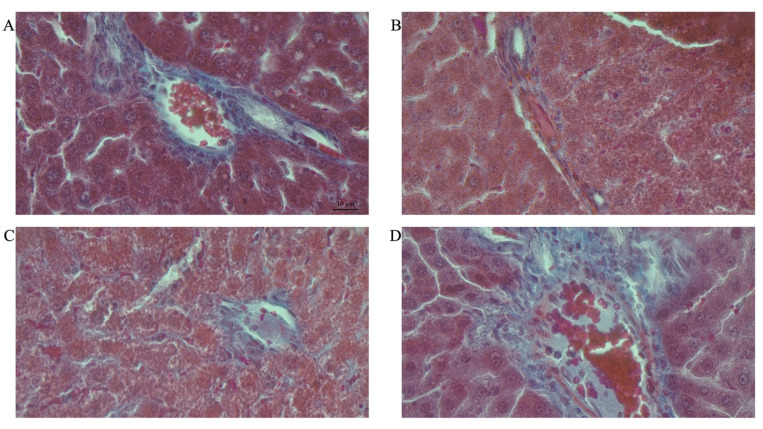

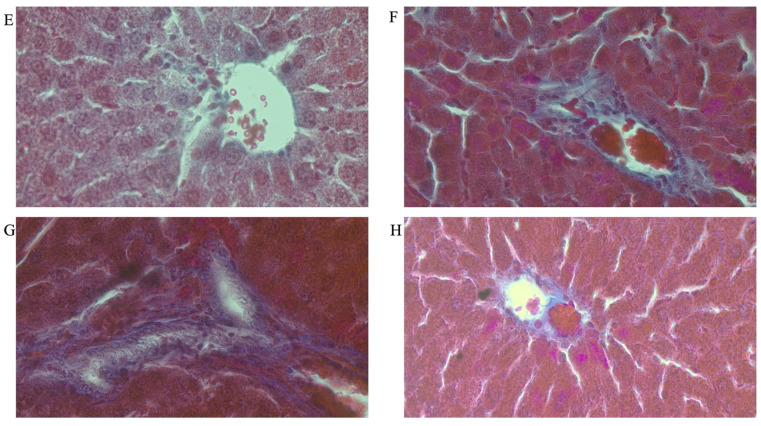
Photomicrographs showing histopathological features after Mason’s trichrome (MT) staining of liver cross-sections from a representative female rat of each experimental group (**A** = Cn + W; **B** = Cn + Fr; **C** = Fr + W; **D** = Fr + Fr; **E** = Chr + W; **F** = Chr + Fr; **G** = Chr Fr + W; **H** = Chr Fr + Fr). Magnification = 40×.

Figure 8A–H shows representative photomicrographs of the liver histology from each of the experimental groups (MT stain) of female rats.

Double-hit (Fr + Fr) high-fructose diets significantly increased (*p* = 0.04 vs. Cn + W; *p* = 0.04 vs. Chr + W) collagen area fraction in the liver of female rats across the various groups (Figure 6F and Figure 8). Finally, late single-hit (Cn + Fr) high-fructose diets significantly increased (*p* = 0.04 vs. Cn + W) collagen area fraction in the liver of female rats across the various groups (Figure 6F and Figure 8). In summary, the high-fructose diet (both single-hit and double-hit) caused a significant increase (*p* < 0.0001) in collagen area fraction, and neonatal chrysin did not prevent the increase.

Overall, there was no significant difference in the percentage collagen area fraction between male and female rats: main sex effects (*p* = 0.0595) and their interaction (*p* = 0.7956). However, there was a significant treatment effect (*p* < 0.0001).

## 5. Discussion

Previous studies have demonstrated the ameliorative effect of chrysin in adult rats [27]. The early life of an organism is characterized by developmental plasticity [28]. Many studies have established the link between neonatal life and the risk of developing NAFLD later in adulthood [29,30]. Therefore, targeting this period for the prevention of metabolic-related diseases, such as NAFLD, is key to curbing the menace later in adulthood [31]. Moreover, we understand that prevention of disease is better than cure. Hence, in this study, we investigated the effect of neonatal oral administration of chrysin on the development of NAFLD in fructose-fed Sprague Dawley rats. 

Non-alcoholic fatty liver disease was quantified by the evaluation of the liver lipids (triglycerides) deposition. The progression and severity of fatty liver disease was assessed by using semi-quantitative non-alcoholic fatty liver disease activity scores (NAS). Increased collagen deposition was observed in rats that received the high-fructose diets (early, late, or double hits), as their area fraction of collagen deposition was significantly higher than that of the control rats. Notably, the timing and frequency of the fructose hit had an impact on the development of NAFLD. Rats that had the high-fructose diets as a single hit (early and late) and double hits recorded a higher percentage of hepatic lipid content when compared with the controls. Using histological analyses, we showed that the liver samples of male and female rats that received the high-fructose diets showed steatosis, inflammation, and fibrosis, which were generally more pronounced in female than male rats, thereby demonstrating sexual dimorphism in the response to the interventions. In the current study, the neonatal administration of chrysin protected male and female rats against some of the component features of NAFLD. Specifically, hepatic lipid accumulation, microsteatosis, hypertrophy, and inflammation were prevented. This is an indication that neonatally administered chrysin could program protection against hepatic steatosis induced by a high-fructose diet.

The hepatosomatic index [32] is influenced by nutritional condition [33] and hepatic pathology, such as fatty liver disease [32]. A severe form of fatty liver disease, such as NASH [32], can cause an increase in the hepato-somatic index, whereas short-term undernutrition can cause a reduction in the hepatosomatic index [33]. We found an increase in intrahepatic lipid accumulation in high-fructose-fed male and female rats, along with a trend to a corresponding increase in hepatosomatic index in the current investigation. This finding is partially consistent with that of Reference [34], who reported that adult male Wistar rats fed a 20% fructose solution for eight weeks developed increased hepatic lipid deposition, but without an increase in liver weight. In our study, the lipid deposition was accompanied by an increased liver mass (hepatosomatic index). This might be a result of the variance in the duration of administration of the fructose diet, as our study was of a longer duration. Moreover, the differences in strain of rat used in both studies could play a role in the discrepancy observed in these findings. It has been reported that Wistar rats are less prone to long-term effects of dietary fructose, due to their active behavior and higher metabolic rate, in contrast to SD rats [35]. 

The hepatosomatic index is increased when the quantity of lipid vacuoles in hepatocytes approaches and exceeds the threshold for causing changes in liver weight [34]. In our study, the steatosis observed was severe enough to induce fibrosis, which is typically linked with a rise in the hepatosomatic index [32]. In female rats, neonatally administered chrysin attenuated the long-term dietary fructose-induced hepatic fat accumulation. It has been reported that chrysin had a lowering effect on liver lipids in adult rats following 8 weeks of oral administration [27]. Our research has demonstrated that a similar attenuating effect could be achieved by strategically targeting the neonatal period. Although hepatic lipid content is important in diagnosing hepatic steatosis, a histological evaluation of hepatocytes is considered a better diagnostic measure for NAFLD [36].

In this study, we observed that an excessive and prolonged fructose diet resulted in microvesicular steatosis (both sexes), macrovesicular steatoses, hypertrophy, and inflammation (female rats only), compared to the control groups. Previous studies in which rats were administered high-fructose diets in the neonatal period have shown the development of micro- and macrovesicular steatosis, hypertrophy, and inflammation [34]. Our report corroborates these findings, as the high-fructose diet also induced NAFLD in the current study. Fructose has been reported to activate the hepatic expression of genes and receptors such as *ChREBP, SREBP-1*, and *PPAR-α* [37], resulting in increased hepatic lipid synthesis and storage [38]. Pai et al. (2019) reported that the oral administration of 100 mg/kg chrysin to adult male Wistar rats brought about a reduced gene expression of *SREBP-1* and an increase in expression of *PPAR-α*, thereby protecting the rats against NASH [27]. Furthermore, it has been suggested that the action of chrysin can also be mediated through its antioxidant and anti-inflammatory properties [27]. 

However, the current study did not investigate the possible mechanisms of action of chrysin at the molecular level of target genes and receptors, and that is acknowledged as a limitation of this investigation.

Undiagnosed and untreated NAFLD can progress to NASH and fibrosis. Collagen maintains the structure of organs [39], and its excessive deposition has been reported to play a major role in development of hepatic fibrosis [39]. An increase in collagen deposition is a biomarker of fibrosis [27]. Excessive fructose intake causes steatosis and subsequent progression to NASH and fibrosis, as indicated by a rise in collagen deposition [40]. This is due to increased levels of free fatty acids (FFAs), which play a significant role in the manifestation of NAFLD [41]. As a result of enhanced lipotoxicity from high levels of FFAs, free cholesterol, and other lipid metabolites, mitochondrial dysfunction with oxidative stress and formation of reactive oxygen species (ROS), as well as endoplasmic reticulum (ER) stress-related processes, are activated [42,43]. 

Additionally, altered gut flora due to diet leads to increased fatty acid production in the intestine, increased small bowel permeability, and, thus, increased fatty acid absorption, as well as increased circulating levels of molecules that contribute to the activation of inflammatory pathways and the release of proinflammatory cytokines such as IL-6 and TNF-α [44]. 

Chrysin has been reported to prevent collagen deposition in adult rats, following 8 weeks of oral administration [27]. However, our study showed that the neonatal administration of chrysin could not prevent the dietary-fructose-induced hepatic fibrosis, thus suggesting a more potent therapeutic effect of chrysin compared to the prophylactic programming effect against hepatic fibrosis.

## 6. Conclusions

A high-fructose diet had an impact on the hepato-somatic index and hepatic lipid accumulation in male and female rats. The high-fructose diet also resulted in the development of specific components of NAFLD, including fibrosis, which were dependent on timing and frequency of the fructose hit. The double-hit high-fructose diet resulted in more severe outcomes than the single-hit, especially in female rats, thereby also expressing sexual dimorphism in the response to the interventions. While neonatal oral administration of chrysin offered some protection against the high-fructose diet induced negative health outcomes, it did not cause any toxicity to the liver of male and female rats. Therefore, the findings of this study have established that the administration of chrysin to neonates may be a viable prophylactic intervention against high-fructose-diet-induced hepatic lipid accumulation and its hepatic sequalae. This can further ease the burden of NAFLD on public and global healthcare facilities. However, further scientific investigations on the gene and receptor interactions may be needed to substantiate this finding.

## Data Availability

The data presented in this study are available on request from the corresponding author.
